# Dietary 2’-Fucosyllactose Enhances Operant Conditioning and Long-Term Potentiation via Gut-Brain Communication through the Vagus Nerve in Rodents

**DOI:** 10.1371/journal.pone.0166070

**Published:** 2016-11-16

**Authors:** Enrique Vazquez, Alejandro Barranco, Maria Ramirez, Agnes Gruart, Jose M. Delgado-Garcia, Maria L. Jimenez, Rachael Buck, Ricardo Rueda

**Affiliations:** 1 Strategic R&D Department, Abbott Nutrition, Granada, 18004, Spain; 2 Strategic R&D Department, Abbott Nutrition, Columbus, OH, United States of America; 3 Division of Neurosciences, Pablo de Olavide University, Seville, 41013, Spain; Universidade de Sao Paulo, BRAZIL

## Abstract

2´-fucosyllactose (2´-FL) is an abundant human milk oligosaccharide (HMO) in human milk with diverse biological effects. We recently reported ingested 2´-FL stimulates central nervous system (CNS) function, such as hippocampal long term potentiation (LTP) and learning and memory in rats. Conceivably the effect of 2´-FL on CNS function may be via the gut-brain axis (GBA), specifically the vagus nerve, and L-fucose (Fuc) may play a role. This study had two aims: (1) determine if the effect of ingested 2´-FL on the modulation of CNS function is dependent on the integrity of the molecule; and (2) confirm if oral 2´-FL modified hippocampal LTP and associative learning related skills in rats submitted to bilateral subdiaphragmatic vagotomy. Results showed that 2´-FL but not Fuc enhanced LTP, and vagotomy inhibited the effects of oral 2´-FL on LTP and associative learning related paradigms. Taken together, the data show that dietary 2´-FL but not its Fuc moiety affects cognitive domains and improves learning and memory in rats. This effect is dependent on vagus nerve integrity, suggesting GBA plays a role in 2´-FL-mediated cognitive benefits.

## Introduction

The richness of HMOs in human milk is unique, not only regarding the total quantity but also considering their diversity and complexity. To date, more than 150 HMOs have been identified [1, 2], being 2’-fucosyllactose (2’-FL) the most abundant HMO, up to 4.65 g/L [3]. On the other hand, oligosaccharides in bovine milk, and therefore in infant formula, are much less abundant and complex [4, 5]. Breastfeeding confers a diverse range of benefits [6, 7]. For example, breastfed children have a higher intelligence quotient, and better performance in intelligence tests later in life compared to those who were formula fed [6, 8].

Learning and memory are mediated by modifications in the strength of synaptic connections between neurons, and the hippocampus is especially important in processes related to information storage and retrieval of memories. Interestingly, the relevance of fucosylated glycoproteins in synaptic junctions and synaptic signaling has been widely described [9, 10]. Furthermore, different studies showed higher radioactively-labelled fucose ([^3^H]Fuc) incorporation to hippocampal proteins after performing different learning paradigms in rats [11] as well as in chicks [12]. LTP is an experimental process through which synaptic strength is rapidly increased, and it has been considered as the key cellular mechanism underlying learning and memory processes [13, 14]. Although the majority of work on LTP has been carried out *in vitro*, LTP has also been observed *in vivo* during learning, and evidence has been provided for the relationships between LTP, activity dependent synaptic plasticity, and associative learning in animals [15]. Individuals with higher and longer-lasting LTP would therefore be expected to exhibit better learning skills. We recently reported ingested 2´-FL enhanced *in vivo* hippocampal LTP and improved learning and memory in new-born[16] and adult[17] rodents. The enhancement in hippocampal LTP induced by both 2’-FL and fucose was also demonstrated in vitro [18] and in freely moving rats after an intrahippocampal injection.

However no information is available about the mechanisms underlying the effect of dietary 2´-FL on brain function. A growing body of evidence highlights the relevance of the gut-brain axis for the appropriate development of many CNS functions [19–21], specifically the vagus nerve as a key pathway in this gut-brain crosstalk. Accordingly it has been shown that vagus nerve stimulation potentiates hippocampal LTP in freely-moving rats [22]. Moreover, Bienenstock et al. showed fucosylated but not sialylated HMOs modulate colon motor contractions, probably by trigering enteric neurons [23]. Pioneering studies demonstrated that [^3^H]Fuc administered intracranially was quickly incorporated into neuronal glycoproteins and transferred to nerve endings suggesting an active process of axonal transport of fucosyl conjugates [24].

Taking this evidence into account, we hypothesized that dietary 2’-FL exerts its modulatory effect on CNS by triggering enteric neurons and those stimuli may reach CNS through the vagus nerve pathway. In this work, we measured *in vivo* hippocampal LTP in rats fed 2´-FL or fucose, as well as the effect of dietary 2´-FL on cognitive skills and hippocampal LTP in rats submitted to bilateral subdiaphragmatic vagotomy.

## Material and Methods

### Experimental animals

**Fig 1:**
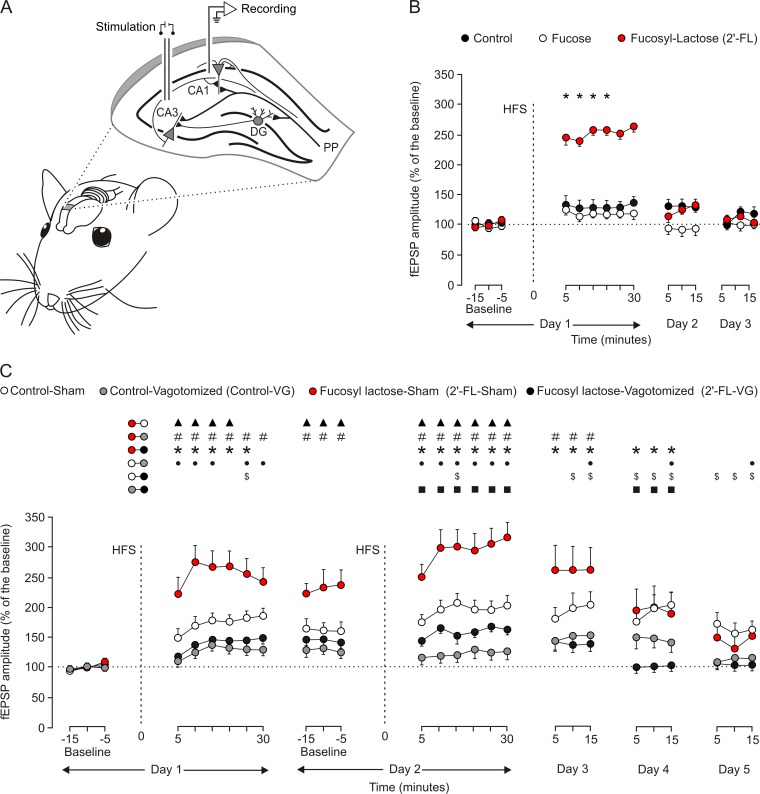
The chronic administration of 2’-FL potentiated LTP evoked at the hippocampal CA3-CA1 synapse in behaving rats, but this positive effect was prevented by a bilateral vagotomy. (A) The diagram illustrated how rats were prepared for the chronic recording of fEPSPs evoked at the hippocampal CA3-CA1 synapse. Bipolar stimulation electrodes were implanted on Schaffer collaterals, while a recording tetrode was aimed at ipsilateral stratum radiatum underneath the CA1 area. (B) A LTP test was carried out in three groups of animals (Control group, black circles; Fucose group, white circles; and 2’-FL group, red circles). After 15 min of baseline records (Day 1) animals were stimulated with a HFS protocol (vertical dotted line). Recording was carried out for 30 min after the HFS protocol. Additional recordings were carried out for 15 min during two additional days (Days 2 and 3). Illustrated data were collected from n ≥ 20 electrodes/group implanted in n ≥ 5 animals/group. Note that the 2'-FL group presented significantly (*, *P* ≤ 0.05) larger LTP values than the other two groups. (C) An additional LTP test was carried out for four groups of animals (Control-Sham group, white circles; Control-Vagotomized group, grey circles; 2’-FL-Sham group, red circles; and, 2’-FL-Vagotomized group, black circles). Animals were stimulated for two successive days with the same HFS protocol. Recording was carried out for 30 min after the two HFS (vertical dotted lines) protocols (Days 1 and 2). Additional recordings were carried out for 15 min during three additional days (Days 3–5). Illustrated data were collected from n ≥ 20 electrodes/group implanted in n ≥ 7 animals/group. Note that the 2'-FL-Sham group presented significantly larger LTP values than the Control-Sham (▲, *P* ≤ 0.05), Control-VG (#, *P* ≤ 0.05), and 2’-FL-VG (*, *P* ≤ 0.05) groups at the indicated recording times. In addition, the control-Sham group presented larger LTP values than the Control-VG (●, *P* ≤ 0.05), and the 2’-FL-VG ($, *P* ≤ 0.05) groups. Finally, the 2’-FL-VG group presented larger LTP values than the Control-VG (■, *P* ≤ 0.05) group for the 2nd recording day, although this situation was reversed during the 4th recording day.

**Fig 2:**
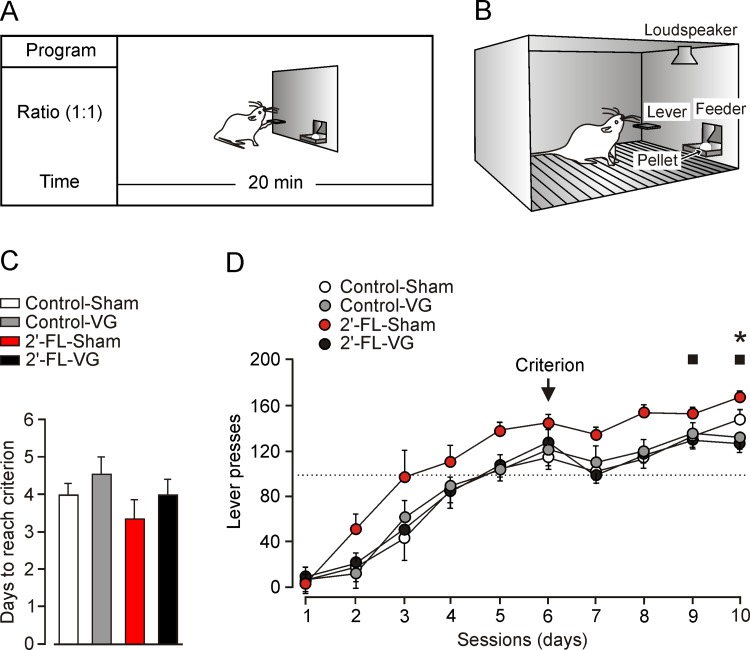
Chronic administration of 2’-FL potentiates the acquisition of an operant conditioning task in behaving rats, but this positive effect was prevented by a bilateral vagotomy. (A,B) Four (Control-Sham, white bar and circles; Control-Vagotomized, grey bar and circles; 2’-FL-Sham, red bar and circles; and, 2’-FL-Vagotomized, black bar and circles) groups of rats (n = 10 per group) were trained to press a lever to obtain a food pellet using a fixed-ratio (1:1) schedule. In this situation, animals have to press the lever just one time to obtain a pellet of food. A tone provided by a loudspeaker indicated the beginning and end of the session. Each session lasted for 20 min. (C) Time to reach criterion (see Methods) for the four experimental groups in the fixed-ratio (1:1) schedule. Although no significant differences were reached between groups, the 2’-FL-Sham group presented a tendency to acquire the task faster than the 2’-FL-Vagotomized (*P* = 0.053) and the Control-Vagotomized (*P* = 0.053) groups. (D) Daily performance in the Skinner box of animals included in the four groups during the fixed-ratio (1:1) schedule. Significant differences were observed between the 2’-FL-Sham group and the 2’-FL-Vagotomized (■, *P* ≤ 0.05) and the Control-Vagotomized (*, *P* < 0.05) groups.

Sprague Dawley male adult rats (2.5–4 months old; 250–300 g) were supplied by Charles River Laboratories. Rats were housed in pairs and kept on a 12 h light/dark cycle with controlled ambient temperature and humidity of 21.5 ± 1°C and 55 ± 8%, respectively. Food and water were available *ad libitum*. Animals devoted to vagotomy as well as to LTP studies were kept individually after surgical procedures. For experiments illustrated in Fig 1B, animals (n = 10 per group) were divided in three groups: Control, Fucose, and 2’-FL. Experiments illustrated in Fig 1C (n = 10 per group) were divided in four groups: Control-Sham, Control-Vagotomized, 2’-FL-Sham, and 2’-FL-Vagotomized. Finally, experiments illustrated in Fig 2 were carried out with additional animals (n = 10 per group) divided in the above mentioned four groups.

This study was carried out in accordance with the guidelines of the European Union (2010/63/UE) and Spanish regulations (RD 53/2013 or BOE-A-2013 1337) for the use of laboratory animals. All experimental protocols were also approved by the local Ethics Committee of Estacion Experimental del Zaidin-CISC (Granada, Spain) as well as for the Ethics Committee of the Servicio de Sanidad Animal, Consejeria de Agricultura y Pesca, Junta de Andalucia (Sevilla, Spain).

### Diets

2´FL and fucose were administered orally through the diet. The control group received a semi purified diet that fit AIN-93M guidelines [25] (manufactured and pelletized by Abbott Laboratories). The 2’-FL and fucose diets were similar to control diet but supplemented with 2’fucosyllactose (2’-FL, acquired from Inalco Pharmaceuticals) at 0.625% (w/w) or fucose (Fuc, acquired from Sigma) at 0.210% (w/w). Dose of 2’-FL in the diet was calculated to provide approximately 350 mg 2’-FL/kg of body weight per day, which was roughly equivalent to the average dose of 2´-FL than breast fed infants would receive. The Fuc dose was calculated to provide equimolar amounts of Fuc and 2´-FL.

### Surgery for bilateral subdiaphragmatic vagotomy

Vagotomy was carried out two weeks before feeding treatments. Rats were anesthetized using gaseous anesthesia with isoflurane using a rodent anesthetics machine. As described in detail previously [26], the stomach and lower esophagus were visualized after an upper midline laparotomy. The skin and abdominal wall were incised along the ventral midline, and the intestine was retracted to allow access to the left lateral lobe of the liver and the stomach. The liver lobule was retracted, and a ligature was placed around the esophagus at its entrance to the stomach to allow gentle retraction to clearly expose both vagal trunks. These were dissected and all neural and connective tissues surrounding the esophagus below the diaphragm were removed to transect all small vagal branches. Sham-operated animals were submitted to the same procedure but vagal trunks as well as connective tissue surrounding the esophagus remained untouched. After surgery, rats received an i.p. injection of penicillin benzatine (1000000 U/rat), and bupremorfine (0,05 mg/kg). At least a 2-wk recovery period was allowed.

### Electrode implantation

Animals used for experiments illustrated in Fig 1 were anesthetized with 4% chloral hydrate at a dose of 1 ml/100 g. Electrode implantation was extensively described in previous articles [15]. Briefly, once anesthetized, animals were implanted with stimulating and recording electrodes in the hippocampus. Stereotaxic coordinates [27] were followed to implant animals with stimulating electrodes aimed at Schaffer collateral-commissural pathway of the dorsal hippocampus (3.5 mm lateral and 3.2 mm posterior to Bregma; depth from brain surface, 1.0–1.5 mm). These electrodes were made of 50 μm, Teflon-coated tungsten wires (Advent Research Materials Ltd., Eynsham, England). In addition, animals were implanted with recording tetrodes aimed at the ipsilateral stratum radiatum underneath the CA1 area (2.5 mm lateral and 3.6 mm posterior to Bregma; depth from brain surface, 1.0–1.5 mm). Recording electrodes were also made of 50 μm Teflon-coated tungsten wires (Advent Research Materials Ltd.). Electrodes were surgically implanted in the CA1 area using as a guide the field potential depth profile evoked by paired (40 ms of interval) pulses presented at the ipsilateral Schaffer collateral pathway. The recording electrodes were fixed at the site where a reliable monosynaptic field excitatory post-synaptic potential (fEPSP) was recorded. A 0.1 mm bare silver wire was affixed to the skull as a ground. The wires were connected to two separate sockets (RS-Amidata, Madrid, Spain). The ground wire was also connected to the recording system with a single wire. Sockets were fixed to the skull with the help of two small screws and dental cement [15].

### Electrophysiological studies

Electrophysiological studies were started 1 week after surgery. As previously described by Gureviviene and coworkers [28], electrophysiological recordings were carried out using Grass P511 differential amplifiers with a bandwidth of 0.1 Hz-10 kHz (Grass-Telefactor, West Warwick, RI, USA). Synaptic field potentials in the CA1 area were evoked by single 100 μs, square, biphasic (negative-positive) pulse applied to Schaffer collaterals. Stimulus intensities ranged from 50 to 350 μA. For each animal, the stimulus intensity was set well below the threshold for evoking a population spike, usually 30–40% of the intensity necessary for evoking a maximum fEPSP response [28]. An additional criterion for selecting stimulus intensity was that a second stimulus, presented 50 ms after a conditioning pulse, evoked a larger (> 20%) synaptic field potential [29].

For evoking LTP, a high frequency (HFS) protocol was used: each animal was presented with five 200 Hz, 100 ms trains of pulses at a rate of 1/s. These trains were presented 6 times in total, at intervals of 1 min. The 100 μs, square, biphasic pulses used to evoke LTP were applied at the same intensity used for evoking baseline records (see [15] for further details of this chronic preparation). Baseline records were collected for 15 min with the paired stimuli presented every 20 s. After the HFS protocol, fEPSPs were recorded again for 30 min. In the case of the experiment illustrated in Fig 1B, additional recordings were carried out for 15 min during the 2 following days. In the case of the experiment illustrated in Fig 1C, one day after the first HFS protocol, we carried out a second baseline (15 min) recording and a second HFS was presented to the experimental animals. After this second HFS, fEPSPs were recorded again for 30 min. Additional recordings were carried out for 15 min during the 3 following days [17].

### Histology to test electrode location

As previously mentioned [17], once the experiments were concluded, rats were deeply re-anesthetized (sodium pentobarbital, 50 mg/kg) and perfused transcardially with saline and 4% phosphate-buffered paraformaldehyde. Selected sections of 50 μm including the dorsal hippocampus were placed on gelatinized glass slides and stained using the Nissl’s staining with 0.1% Toluidine blue, in order to confirm the correct location of stimulating and recording electrodes.

### Operant conditioning of rats

Animals received experimental diets for five weeks. According to the procedure previously described [17], training and testing took place in basic Skinner box modules (n = 5) measuring 29.2 × 24.1 × 21 cm (MED Associates, St. Albans, VT, USA). The operant chambers were housed within a sound-attenuating chamber (90 × 55 × 60 cm), which was constantly illuminated (19 W lamp) and exposed to a 45 dB white noise (Cibertec, S.A.). Each Skinner box was equipped with a food dispenser from which pellets (MLabRodent Tablet, 45 mg; Test Diet, Richmond, IN, USA) could be delivered by pressing a lever. Before training, rats were handled daily for 7 days and food-deprived to 80–85% of their free feeding weight until the end of the operant conditioning test. Once the desired weight was reached, animals were placed in the Skinner box for 20 min and allowed to press the lever to receive pellets from the food tray using a fixed-ratio (FR 1:1) schedule, until reaching criterion. The selected criterion was to press the lever up to 40 times/session and to repeat the same rate during the following session. Animals were allowed a maximum of 10 days to reach criterion. The start and end of each session was indicated by a tone (2 kHz, 200 ms, 70 dB) provided by the loudspeaker located in the recording chamber (Fig 2A and 2B). Conditioning programs, lever presses, and delivered reinforcers were controlled and recorded by a computer, using a MED-PC program (MED Associates).

### Statistical analysis

The normality of data distribution and homogeneity of variances were confirmed in each set of data before applying the different statistical tests. Hippocampal activity and 1-volt rectangular pulses corresponding to stimulus presentations were stored digitally on a computer through an analog/digital converter (CED 1401 Plus), at a sampling frequency of 11–22 kHz and an amplitude resolution of 12 bits. Commercial computer programs (Spike 2 and SIGAVG from CED) were modified to represent extracellular synaptic field potential recordings. The slope of evoked fEPSPs was collected as the first derivative (i.e., mV/s) of fEPSP records (mV). For this, 5 successive evoked field synaptic potentials were averaged, and the mean value of the slope was determined for the rise time period (i.e., the period of the slope between the initial 10% and the final 10% of the evoked field potential). Unless otherwise indicated, data are presented as the mean value collected from each experimental group followed by the SEM. Graphic displays were constructed with the help of the SPSS package (SPSS Inc, Chicago, IL, USA). Statistical differences between groups were determined with a two-way repeated means of the analysis of variance (ANOVA), the session (days) or the trial being the repeated means factor. Statistical differences between two groups were studied with all pairwise multiple comparison procedures (Holm-Sidak method). The significance level was established at P = 0.05 for all tests. We applied the Mann-Whitney test in Fig 2*C* because the data did not accomplish the normality test. These statistical procedures have been described in detail elsewhere [15, 30].

## Results

### Chronic oral administration of 2’-FL but not fucose improves LTP

As described in the Methods section, LTP was evoked in the hippocampal CA1 area originated from ipsilateral CA3 pyramidal cells (Fig 1A). The evolution of fEPSPs evoked at the CA3-CA1 synapse by single pulses presented before and after an HFS session was followed in three groups of animals (n = 10 animals per group; Fig 1*B*): Control, Fuc and 2’-FL. Stimulus intensity was set at 35% of the intensity necessary for evoking a maximum fEPSP response for both HFS and for baseline and post-HFS recordings. Changes in fEPSPs evoked at the hippocampal CA3-CA1 synapse following the HFS protocol were followed for up to three days [*F*_(28,252)_ = 5.08; *P* < 0.05; two way repeated measures analysis]. The Control and Fuc groups showed similar LTP values during all the study (*P* ≤ 0.426). On the other hand, the 2'-FL group presented significantly larger (*P* ≤ 0.05, see asterisks in Fig 1B) LTP values than the other two (Fuc, Control) groups during the 1st post-HFS session. Chronic oral administration of intact 2’-FL caused a larger and longer-lasting LTP response at the hippocampal CA3-CA1 synapse, whereas these potentiation effects could not be evoked by Fuc.

### Vagotomy inhibits the effect of 2’-FL on LTP

Experiments were designed to determine if the potentiating effects of chronic administration of 2’-FL on LTP were dependent on vagal innervation of the intestine. Experimental animals (n = 10 per group) were divided in four groups: Control-Sham, Control-Vagotomized, 2’-FL-Sham, and 2’-FL-Vagotomized. In order to reach larger LTP levels, rats were presented in this case with two successive HFS sessions (separated 24 h; Fig 1C). Changes in fEPSPs evoked at the hippocampal CA3-CA1 synapse following the two HFS protocols were followed for up to three days after the second HFS session [*F*_(78,702)_ = 4.712; *P* < 0.05; two way repeated measures analysis]. The 2'-FL-Sham group presented significantly larger (*P* ≤ 0.05) LTP values than the other three groups. Vagotomy produced a significant (*P* ≤ 0.05) reduction in the evoked LTP in both Control-Vagotomized and 2’-FL-Vagotomized groups compared to Control group. Interestingly, the effects of vagotomy were more evident in the Control-VG than in the 2’-FL-VG group, with significant (*P* < 0.05) differences for the 2nd and 4th days (see Fig 1C).

### Vagotomy prevents potentiating effects of 2’-FL on operant conditioning

Fig 2A *and 2B* shows the experimental design for the acquisition of an operant conditioning task. In short, rats (n = 10 per group) were trained in 5 Skinner boxes to press a lever in order to obtain a small pellet of food, using a (FR 1:1) schedule (i.e., a food pellet following each lever press). All of the experimental animals acquired the operant conditioning task with the fixed-ratio schedule. The selected criterion was to press the lever 40 times/session for two successive 20-minute sessions (see Material and Methods). Interestingly (Fig 2C), animals included in the 2’-FL-Sham group reached the selected criterion in less (3.41 ± 0.36; *P* = 0.053; Mann-Whitney test) sessions than the 2’-FL-Vagotomized (4.0 ± 0.43), the Control-Vagotomized (0.59 ± 0.41), and the Control-Sham (4.0 ± 0.27) groups. In addition, the 2’-FL-Sham group presented a better performance (indicated by the mean number of lever presses per session) than the other three groups across the ten sessions of the fixed-ratio (FR 1:1) schedule [*F*_(27,243)_ = 3.224; *P* < 0.05; two way repeated measures analysis; Fig 2D]. Thus, by the 10th training session, the 2’-FL-Sham group pressed the lever a mean of 166.1 ± 2.2 times, an amount significantly larger (*P* ≤ 0.05) than mean lever presses carried out by the 2’-FL-Vagotomized (n = 123.3 ± 2.2), the Control-Vagotomized (n = 125.5 ± 3.3). But, no significant differences were reached between the 2’-FL-Sham and the Control (n = 142.7 ± 4.4) groups. In conclusion, and as previously reported [17], the administration of 2’-FL improved cognitive performance in the Skinner box. Nevertheless, and in accordance with the present results these facilitating effects were eliminated by a bilateral vagotomy.

## Discussion

The benefits of breast milk for the newborn are well established. Several studies, including a meta-analysis, have confirmed that breastfed infants develop higher intelligence quotients and perform better in intelligence tests than formula-fed infants. Differences in the composition of human and bovine milk may be responsible for such differences. HMOs have been postulated as key ingredients for brain development due to their high abundance and variety in maternal milk when compared to bovine-milk based formulas [31]. Fucosylated HMOs are predominant in breast milk, especially 2’-FL which is the most abundant HMO, in most women’s milk [32]; while oligosaccharides from cow’s milk are mainly sialylated, and are present at low levels. Our group we reported the first *in vivo* study of dietary HMOs on CNS function, whereby 2´-FL potentiated LTP evoked at the hippocampus, improved learning and memory skills and impacted several brain markers of synaptic plasticity and brain function in rodents [17]. Recently, we also demonstrated that 2´-FL positively modulates cognition not only in adult rodent but also in young rats (Oliveros et al., 2016). Tarr et al (2015) recently showed other HMOs (3’- and 6’-sialylactose) reduce stressor-induced anxiety-like behavior, confirming the impact of select HMOs on cognitive function. These data support the role of HMOs in signaling crosstalk between the intestine and brain through different pathways of the GBA [33].

The concept that 2´-FL or its Fuc moiety may affect synapses, and therefore CNS function, is not novel. A relatively old report [34] indicated that intrahippocampal administration of L-fucose or 2’-FL had a potentiating effect on LTP evoked at the perforant pathway-dentate gyrus synapse in freely moving rats. Another study reported both molecules increased the potentiation of the population spike amplitude and fEPSP after LTP induction in the CA3-CA1 in hippocampal slices from rats [18]. Conversely, L-fucose and 3-FL had no potentiating effects on experimentally evoked LTP [18]. There are several articles describing some mechanisms that may be partially responsible for the effects of 2´-FL or Fuc on synaptic function [18, 34]. Injected L-fucose may have an influence on glutamate release at the hippocampal CA3-CA1 synapse [35]. Moreover, glycosylation of proteins during a critical time window following HFS of hippocampal slices is necessary for the adequate induction as well as maintenance of the evoked LTP [36], demonstrating the importance of Fuc residues in the frame of adequate neuronal processing. Similarly, there is evidence supporting a role for Fuc and fucosyl derivatives in maintenance of synaptic function. Murray et al. (2006) reported protein fucosylation regulates the expression of key genes, such as synapsin Ia/Ib, and neuronal morphology in hippocampal neurons [37] and also described the importance of other fucosylated sugars, such as fucose-alpha(1–2)-galactose, in neuronal plasticity in mouse olfactory bulbs [10]. However, those data were obtained by adding the compounds intracranially or incorporated in the culture medium. To our knowledge there was no published data about the role of dietary Fuc or fucosylated compounds, on the regulation or maintenance of CNS synapses, although some studies pointed out Fuc as an important mediator of host-microbe symbiosis under normal [38] or pathological scenarios [39]. This may imply Fuc as a potential compound for GBA activation. The current study elucidated the role of intact 2’FL vs. Fuc on CNS functions. The reason why we chose as control Fuc instead of lactose is that, based on the literature mentioned above, Fuc was a much better candidate than lactose for being an active compound in the brain. We wanted to guaranteed that the effects of 2´-FL on LTP was not related to the fact that 2´-FL may be a Fuc source. However, our results showed that orally administered Fuc did not alter hippocampal LTP values compared to control rats, whereas 2´-FL induced a significant facilitatory effect, confirming previous results [17]. Thus, ingested L-fucose exerted no effect on LTP, in contrast to previous reports in which it was exogenously added to brain slices or injected into the hippocampus. This suggests molecular integrity of 2´-FL at the intestine is necessary to induce beneficial effects on CNS function, although the specific GBA pathway responsible for such effects is unknown.

The peripheral components of the GBA connect with the central nervous system through several channels such as the enteric, autonomic, and sympathetic nervous systems [33, 40]. A growing body of evidence states gut microbes may modulate brain development and function [41, 42] via the enteric nervous system (ENS). The ENS resides within the intestinal wall and abnormalities in the ENS are associated with a wide spectrum of gastrointestinal disorders in both adults [43] and infants [33]. The ENS networks with the brain via the vagus nerve and dorsal root as well as nodose ganglia [40], and the afferent vagus nerve is the major retrograde signaling system from gut to brain [44]. Evidence for the latter was provided by the ingestion of a *Lactobacillus* strain shown to regulate emotional behavior and central GABA receptor expression due to vagus nerve integrity [26]. Furthermore vagus nerve stimulation potentiates hippocampal LTP in freely-moving rats [22].

Recent reviews highlight the importance of the relationship between the microbiome and ENS regulation [19, 41]. The prebiotic character of HMOs and their speculated role for the maintenance of an adequate microbial community in the newborn intestine is one of the most referenced properties of HMOs [45]. HMOs resist digestion processes and serve as preferred metabolic substrates for select microbes, contributing to the infant gut microbiome [46]. Variations to the balance of common intestinal microorganisms might modify production of the short-chain fatty acids, which are products of intestinal bacterial fermentation that play key roles in CNS function [47, 48]. HMOs have been also reported as factors inducing gut maturation [49] as well as preventing pathological aberrations of the intestinal mucosa as severe as necrotizing enterocolitis [50]. Thus, HMOs play a regulatory role at the microbe-intestinal mucosa interface. HMOs also may activate ENS constituents residing within the intestinal wall. In this sense, it has been reported that fucosylated HMOs (such as 2´-FL) diminish colon motor contractions in an *ex vivo* model [23], revealing a direct stimulus of enteric nerves by fucosylated HMOs, although the authors also suggest they may stimulate the brain via the vagus nerve.

Based on the evidence, we hypothesized the afferent vagus nerve could be involved in the modulatory effect of ingested 2´-FL on brain function. In this study, we confirmed this hypothesis, whereby bilateral vagotomy prevented the facilitating effect of 2´-FL on LTP (Fig 1C). Likewise, sham-operated animals fed with a 2´-FL supplemented diet exhibited a potentiated LTP (Fig 1C) as well as better performance in associative learning related tests (Fig 2C and 2D), in accordance with previously reported data [17]. Disruption of the vagal pathway led those learning scores of vagotomized rats fed on 2´-FL animals to those from control rats. Although our research on LTP focused on the hippocampus, ingested 2’-FL also affected operant conditioning, suggesting other neuronal circuits outside of the hippocampus may be involved [51]. Therefore, it is highly possible that 2´-FL modulates different brain areas by triggering enteric neurons and, excitatory signals may reach the brain through the vagal pathway of the GBA.

Another interesting effect we found in our study is that control animals subjected to bilateral subdiaphragmatic vagotomy exhibited a weaker LTP than Sham-operated rats. Severing of the peripheral axons of the vagus nerve may have side-effects in the CNS. Subdiaphragmatic vagotomy is known to trigger remodeling in the nucleus tractus solitaries (NTS). Vagotomy synapses in the NTS were significantly reduced 10 days following vagotomy but were restored to control levels by 30 days and 60 days. Electrophysiology also revealed transient decreases in spontaneous glutamate release, and the number of primary afferent inputs [52]. Other changes in the CNS induced by vagotomy, such as microglial activation within the hindbrain, nodose ganglia, and the spinal cord have also been described [53]. However, to our knowledge, no data about the potential role of subdiaphragmatic vagotomy on LTP has been previously described. However, it is worthy to point out that, considering our results; the effects of vagotomy were more evident in the Control-VG than in the 2’-FL-VG group. The weak enhancing effect of 2´-FL on LTP in vagotomized animals is sufficient to be detected in our electrophysiological experiments, but not strong enough to affect performance in associative learning related tests. This suggests that, although the vagus nerve appears to be the main pathway underlying the potentiating effects of 2´-FL on CNS functions, and taking into account the high complexity of the GBA network, it may also exit a secondary pathway by which 2´-FL may slightly modulate CNS in animals with the vagus nerve disrupted. On regards to this, it has been reported that a relatively small amount of intact HMOs is absorbed from the intestine into systemic circulation after oral administration. This phenomenon has been described in animal models using neonatal pups [54] and adult rats [55] as well as in breastfed infants [56]. Thus, HMOs present in the bloodstream may, potentially, reach the brain and somehow modulate CNS function. However, this potential systemic pathway of HMOs remains to be elucidated. Further investigation is needed to confirm this complementary hypothesis. Finally, we cannot exclude neither that the prebiotic function of HMOs may also indirectly modulate in some way this gut-brain crosstalk. Since HMOs are able to shape the microbial communities at the intestinal ecosystem, the levels of some products derivate from bacterial metabolism such as short chain fatty acids, peptides, neurotransmitters, etc. able to activate GBA may be also modified, and therefore play a potential role in this effect of 2´-FL on CNS function. Further research is needed in order to clarify these potential secondary routes by which HMOs may modulate the gut-brain crosstalk. Additionally, since some reports about the differences in response between male and female rodents in the frame of GBA have being recently published, in future research, animals from both genders should be included.

### Conclusion and final remarks

In summary, orally administered 2’FL enhances brain function and cognition via the vagus nerve in rats. Ingested Fuc did not exert these effects, although the Fuc moiety can enhance LTP and learning/memory abilities via intraventricular or intraperitoneal injection. Ingested Fuc exerted no effect on synapsis regulation, suggesting that molecular integrity of 2’FL at the intestine is necessary to induce beneficial effects on CNS function. Furthermore the afferent vagus nerve pathway is the major route for transmitting enteric signals generated by the presence of 2´-FL from the intestine to the brain.

Despite the fact that 2’-FL is present in urine [57, 58] and plasma [56] of breastfed babies, and is absorbed and transported through the bloodstream without modification, there is no published report confirming the presence of 2’-FL in the brain nor a mechanism to cross the blood-brain barrier. Subsequently, we elucidated the GBA, and more specifically, vagus nerve connections are involved in the mechanism of action by which dietary 2’-FL enhances cognitive abilities in rodent models.
